# Phylogeography and Population Genetics of *Vicugna vicugna*: Evolution in the Arid Andean High Plateau

**DOI:** 10.3389/fgene.2019.00445

**Published:** 2019-06-06

**Authors:** Benito A. González, Juan P. Vásquez, Daniel Gómez-Uchida, Jorge Cortés, Romina Rivera, Nicolas Aravena, Ana M. Chero, Ana M. Agapito, Valeria Varas, Jane C. Wheleer, Pablo Orozco-terWengel, Juan Carlos Marín

**Affiliations:** ^1^Laboratorio de Ecología de Vida Silvestre, Facultad de Ciencias Forestales y de la Conservación de la Naturaleza, Universidad de Chile, Santiago, Chile; ^2^South American Camelid Specialist Group, Survival Species Commission, International Union for Conservation of Nature, Santiago, Chile; ^3^Laboratorio de Genómica y Biodiversidad, Departamento de Ciencias Básicas, Facultad de Ciencias, Universidad del Bío-Bío, Chillán, Chile; ^4^GEECLAB, Departamento de Zoología, Facultad de Ciencias Naturales y Oceanográficas, Universidad de Concepción, Concepción, Chile; ^5^Núcleo Milenio INVASAL, Concepción, Chile; ^6^Departamento de Ciencias Básicas, Facultad de Ciencias, Universidad Santo Tomás, Iquique, Chile; ^7^Doctorado en Ciencias, Mencioìn Ecologiìa y Evolucioìn, Instituto de Ciencias Ambientales and Evolutivas, Facultad de Ciencias, Universidad Austral de Chile, Valdivia, Chile; ^8^CONOPA-Instituto de Investigación y Desarrollo de Camélidos Sudamericanos, Lima, Peru; ^9^School of Biosciences, College of Biomedical and Life Sciences, Cardiff University, Cardiff, United Kingdom

**Keywords:** camelids, vicuña, d-loop, microsatellites, subspecies

## Abstract

The vicuña (*Vicugna vicugna*) is the most representative wild ungulate of the high Andes of South America with two recognized morphological subspecies, *V. v. mensalis* in the north and *V. v. vicugna* in the south of its distribution. Current vicuña population size (460,000–520,000 animals) is the result of population recovery programs established in response to 500 years of overexploitation. Despite the vicuña’s ecosystemic, economic and social importance, studies about their genetic variation and history are limited and geographically restricted. Here, we present a comprehensive assessment of the genetic diversity of vicuña based on samples collected throughout its distribution range corresponding to eleven localities in Peru and five in Chile representing *V. v. mensalis*, plus four localities each in Argentina and Chile representing *V. v. vicugna.* Analysis of mitochondrial DNA and microsatellite markers show contrasting results regarding differentiation between the two vicuña types with mitochondrial haplotypes supporting subspecies differentiation, albeit with only a few mutational steps separating the two subspecies. In contrast, microsatellite markers show that vicuña genetic variation is best explained as an isolation by distance pattern where populations on opposite ends of the distribution present different allelic compositions, but the intermediate populations present a variety of alleles shared by both extreme forms. Demographic characterization of the species evidenced a simultaneous and strong reduction in the effective population size in all localities supporting the existence of a unique, large ancestral population (effective size ∼50,000 individuals) as recently as the mid-Holocene. Furthermore, the genetic variation observed across all localities is better explained by a model of gene flow interconnecting them rather than only by genetic drift. Consequently, we propose space “continuous” Management Units for vicuña as populations exhibit differentiation by distance and spatial autocorrelation linked to sex biased dispersal instead of population fragmentation or geographical barriers across the distribution.

## Introduction

The vicuña (*Vicugna vicugna*) is the most representative wild ungulate of the Andean high plateau in South America (Franklin, 1983; Wheeler, 1995, 2012). Its current distribution is limited to extreme altitude environments, living in arid landscapes with intense solar radiation and a hypoxic atmosphere (Franklin, 1982). Two morphological subspecies have been described, with geographical and habitat differences and supported by mitochondrial DNA markers (Marín et al., 2007a,b; Casey et al., 2018) the northern vicuña (*V. v. mensalis*) and the southern vicuña (*V. v. vicugna*). The subspecies *mensalis*, inhabits the ‘moist puna,’ is smaller and darker than the southern vicuña and is distinguished primarily by the long growth of hair on the chest (Wheeler, 2012). In contrast, the subspecies *vicugna* inhabits ‘dry puna’ within the Dry Diagonal belt (24° and 29° S; Ammann et al., 2001; Kull et al., 2002), lacks the long chest hairs, and has a lighter beige pelage coloration with white covering a greater portion of the body, rising halfway up the sides to mid-rib height and all the way to the ileum crest (Wheeler, 2012). Finally, the greater length of the southern vicuña molar line supports phenotypic differentiation (Wheeler and Laker, 2009). This division into two groups is further supported by the presence of two mitochondrial lineages differentiating each subspecies (Marín et al., 2017), with the southern subspecies showing greater haplotypic diversity than the northern one (Marín et al., 2007b). The biogeographical barrier between the subspecies has been suggested to correspond to the deep valley of Tarapaca in Chile (19°S) on the western side of the vicuña distribution, however, there is no evident barrier at a similar latitude on the eastern side (Bolivia) of the species distribution range (Wallace et al., 2010). Current vicuña distribution covers an area of 300,000 km^2^ with several populations having increased their numbers after a drastic historic reduction (Acebes et al., 2018). Distribution is limited to altitudes from ∼3,000 to ∼5,000 m above sea level (Baigún et al., 2008; Villalba et al., 2010) along a 2,600 km stretch of the Central High Andes between 9° 30′ S in Ancash, Peru, and 27° 31′ S in the San Guillermo Reserve, Argentina (Wheeler and Laker, 2009). During the 1980s Chile, Peru, and Bolivia donated vicuña to Ecuador which were introduced to Chimborazo National Park (1° 31′ S, 78° 51′ W) and currently represent a stable, growing population (Rodriìguez and Morales-Delanuez, 2017; Vicuña Convention, 2017). This population should not be considered part of the natural vicuña distribution as there is no sound evidence that the species previously existed in Ecuador.

Current vicuña distribution and abundance is the result of population recovery programs established in response to 500 years of overexploitation (Yacobaccio, 2009) and near extinction in the 1960s (Wheeler and Laker, 2009). At the time of lowest population size only 6,000–10,000 vicuñas were left, widely distributed in low density, highly dispersed populations, with some small groups persisting at the species’ southern distribution range (Grimwood, 1969; Boswall, 1972; Jungius, 1972). Thanks to the establishment of national parks and reserves, the Andean Vicuña Convention agreement, and funds from international NGOs, the vicuña population notably increased to over 200,000 individuals in four decades (Wheeler, 2006), with the northern populations showing greater recovery than the southern ones (Wheeler and Laker, 2009). Currently, a total population about 460,000–520,000 individuals inhabit the Andean high plateau (Vicuña Convention, 2017; Acebes et al., 2018), corresponding to a 50-fold increase in five decades of intensive protection and management.

Studies of vicuña genetics are limited and geographically restricted. Genetic structure has been determined using both nuclear and mitochondrial DNA in Peruvian localities (Wheeler et al., 2001), the north of Chile and Bolivia (Sarno et al., 2004), and north-western Argentina (Anello et al., 2016). The results of these studies have been used to identify four discrete Management Units (MUs; Wheeler et al., 2001; Casey et al., 2018) for the maintenance of locally adapted populations in Peru. MUs are defined as demographically independent populations whose dynamics depend on local birth and death rates rather than immigration from other populations (Taylor and Dizon, 1999). Although several researchers have advocated the use of MUs (e.g., Mosa, 1987; Marín et al., 2013b; Moodley et al., 2017), this approach has not been used for the vicuña despite its ecological, cultural and conservation importance (Wheeler et al., 2001; Sarno et al., 2004). Practical aspects deriving from the implementation of such classification would facilitate determination of the origin of skin and fiber from confiscated illegally hunted and trafficked materials (González et al., 2016), as has been done for other species (Moodley et al., 2017). Additionally, a thorough characterization of the vicuña genetic variation would facilitate comparison between the genetic diversity of wild populations and managed, captive production groups (Stølen et al., 2009; Escalante et al., 2014; Anello et al., 2016).

Here, we present a comprehensive assessment of the molecular diversity of vicuña based on samples collected throughout its distribution range. We analyze their genetic variation using 15 microsatellite loci and sequences of the left domain of the mitochondrial control region. We present evidence of (1) range-wide phylogeographic structure linked with vicuña evolutionary history; (2) patterns of molecular genetic structure among vicuñas; and (3) links between patterns of genetic variation with phylogeographic history and barriers to gene flow. We further utilize this evidence to (4) describe and contrast the evolutionary history and patterns of gene flow among these populations in order to propose effective MUs for the species at broad scale.

## Materials and Methods

### Ethics Statement

**Figure 1:**
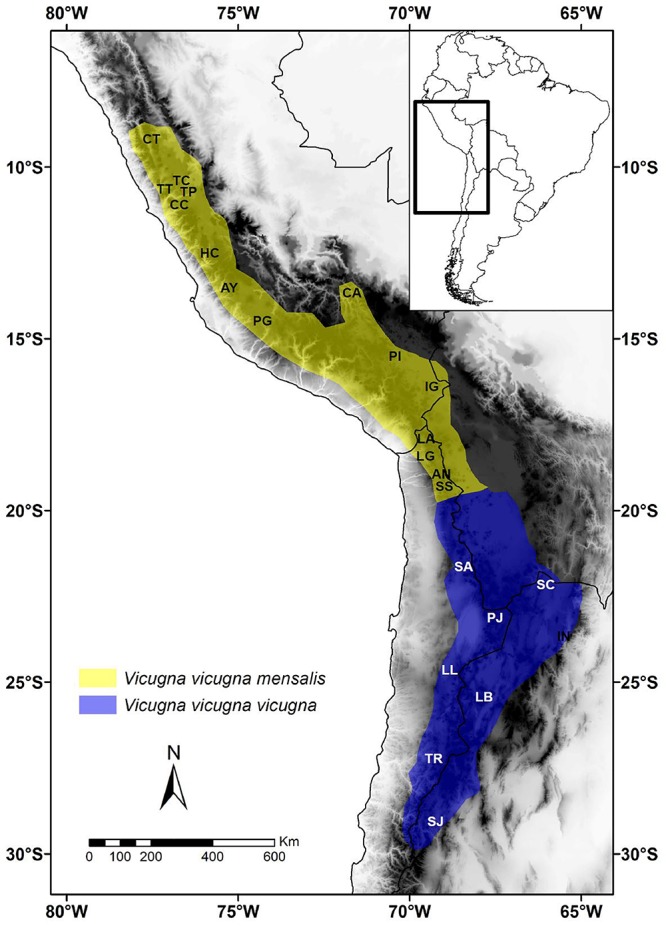
Map of the geographic distribution of *Vicugna vicugna mensalis* (yellow) and *V. v. vicugna* (blue) and location of sampled populations in South America. Localities names are: Catac (CT), Tinco Cancha (TC), Tinco Paccha (TP), Tarma Tambo (TT), Sto. Domingo de Cachi Cachi (CC), San Pedro de Hacarpana (HC), Ayavi-Tambo-Huaytará (AY), R. N. Pampa Galeras (PG), Cerro Azul (CA), S.A.I.S. Picotani (PI), Ingenio Hacullani (IG), Parque Nacional Lauca (LA), Lagunillas (LG), Corral Ankara (AN), Salar de Surire (SS), Salar de Ascotán (SA), Paso Jama (PJ), P. N. Llullaillaco (LL), P. N. Nevado Tres Cruces (TR), Santa Catalina (SC), INTA Abra Pampa (IN), Laguna Blanca (LB), San Juan (SJ) (Table 1).

Samples were collected throughout the current distributional range of the vicuña (Table 1 and Figure 1) following guidelines of the American Society of Mammalogists (Sikes et al., 2011). Specific permits were required for the Servicio Agrícola y Ganadero, SAG (permit 447, 2002), the Corporación Nacional Forestal, CONAF (permit 6/02, 2002), for granting other collection permits and helping in collecting samples. The animal research oversight committee of Universidad del Bío-Bío had knowledge of sampling plans prior to their approval of the present animal research protocol. All experimental protocols were approved by the Institutional Animal Care and Use Committee of Universidad del Bío-Bío, the methods were carried out in accordance with the approved guidelines. Samples were collected and exported for analysis (CITES permits 6282, 4222, 6007, 5971, 0005177, 0005178, 023355, 022967, and 022920) and imported to the United Kingdom (permits 269602/01, 262547/02). Peruvian samples were collected under permits from CONACS (28 September 1994, 15 June 1997), INRENA (011-c/c-2004-INRENA-IANP; 012-c/c-2004-INRENA-IANP; 016-c/c-2004-INRENA-IFFS-DCB; 016-c/c-2004-INRENA-IFFS-DCB; 021-c/c-2004-INRENA-IFFS-DCB; 026-c/c-2005-INRENA-IANP) and DGFFS (109-2009-AG-DGFFS-DGEFFS).

**Table 1:** Summary of the *Vicugna vicugna* samples, including localities (ordered north to south), sample type (B, blood; F, fecal; M, muscle; S, skin), number of samples analyzed from each locality for each genetic marker.

Region	Localities; Country (abbreviation)	Sample type	Samples mtDNA (*N* = 353)	Samples microsatellites (*N* = 307)
**Northern region**		–	241	179
	Catac, Ancash; Perú (**CT**)	B	14	11
	Tinco Cancha, Junín; Perú (**TC**)	B	16	6
	Tinco Paccha, Junín; Perú (**TP**)	B	16	5
	Tarma Tambo, Junín; Perú (**TT**)	B	8	8
	Sto. Domingo de Cachi Cachi, Junín; Perú (**CC**)	B	12	8
	San Pedro de Huacarpana, Ica; Perú (**HC**)	B	17	7
	Ayavi-Tambo-Huaytará, Huancavelica; Perú (**AY**)	B	7	8
	R. N. de Pampa Galeras, Ayacucho; Perú (**PG**)	B	21	15
	Cerro Azul, Cuzco; Perú (**CA**)	B	27	17
	S.A.I.S. Picotani, Puno; Perú (**PI**) (captivity)	B	10	7
	Ingenio Huacullani, Puno; Perú (**IG**)	B	16	16
	
	Parque Nacional Lauca; Chile (**LA**)	S, B	34	29
	Lagunillas; Chile (**LG**)	B	16	16
	Corral Ankara, Ankara; Chile (**AN**)	B	10	9
	Salar de Surire; Chile (**SS**)	B	17	17

**Southern region**		–	112	128
	Salar de Ascotán; Chile (**SA**)	F	17	28
	Paso Jama; Chile (**PJ**)	F	5	4
	P. N. Llullaillaco; Chile (**LL**)	B	9	10
	P. N. Nevado Tres Cruces; Chile (**TR**)	M, F, S	9	11
	
	Santa Catalina, Jujuy; Argentina (**SC**)	B	21	21
	INTA Abra Pampa, Jujuy; Argentina (**IN**) (captivity)	B	28	33
	Laguna Blanca, Catamarca; Argentina (**LB**)	B	20	18
	San Juan; Argentina (**SJ**)	B	3	3

### Sample Collection and DNA Extraction

Three hundred and fifty-three samples were collected between 1994 and 2004 at eleven Peruvian and five Chilean localities currently designated as *V. v. mensalis;* as well as four Argentine and four Chilean localities currently designated as *V. v. vicugna* (Figure 1 and Table 1). Samples comprised skin (*n* = 4), muscle (*n* = 2), blood (*n* = 333), and feces (*n* = 37). All samples were stored at -80°C in the Laboratorio de Genómica y Biodiversidad, Departamento de Ciencias Básicas, Facultad de Ciencias, Universidad del Bío-Bío, Chillán, Chile or at CONOPA in Lima, Peru. Total genomic DNA was extracted from blood using the Wizard Genomic DNA Purification Kit (Promega, Madison, WI, United States). DNA from skin and muscle samples was purified using proteinase K digestion and a standard phenol–chloroform protocol (Sambrook et al., 1989). DNA from feces was extracted using the QIAamp DNA Stool Mini Kit (QIAGEN, Valencia, CA, United States) in a separate non-genetic-oriented laboratory.

### Mitochondrial DNA

Three hundred and eighty-five base pairs of the left domain of the mitochondrial control region (mtDNA-CR) was amplified using the camelid and vicuña-specific primers LthrArtio (5′- GGT CCT GTA AGC CGA AAA AGG A-3′), H15998V (5′-CCA GCT TCA ATT GAT TTG ACT GCG-3′), Loop007V (5′-GTA CTA AAA GAG AAT TTT ATG TC-3′), H362 (5′-GGT TTC ACG CGG CAT GGT GAT T-3′) (Marín, 2004). Amplification was performed in 50 ml with ∼30 ng genomic DNA, 1x reaction buffer (8 mM Tris-HCl (pH 8.4), 20 mM KCl (Invitrogen, Gibco, Life Technologies, Invitrogen Ltd., Paisley, United Kingdom), 2 mM MgCl_2_, 25 μM each of dNTP, 0.5 μM each primer and 0.1 U/μl Taq polymerase (Invitrogen, Gibco, Life Technologies). Thermocycling conditions were: 95°C for 10 min, followed by 30–35 cycles of 94°C for 45 s, 62°C for 45 s, 72°C for 45 s, then 72°C for 5 min. PCR products were purified using the GeneClean Turbo for PCR Kit (Bio101) following the manufacturer’s instructions. Products were sequenced in forward and reverse directions using BigDye chemistry on an ABI Prism 3100 semiautomated DNA analyzer, and consensus sequences were generated and aligned using Geneious v.9.1.5 (Biomatters, Auckland, New Zealand). The final alignment was trimmed to 328 bp beginning at the 5′ left domain of the d-loop.

The number of segregating sites (*S*) and haplotypes (*nh*), haplotype diversity (*h*) (Nei, 1987), nucleotide diversity (π) and average number of nucleotide differences between pairs of sequences (*k*) were estimated using ARLEQUIN 3.5.1.2 (Excoffier and Lischer, 2010). A statistical parsimony network was constructed using TCS v1.21 (Clement et al., 2000) with default settings.

### Microsatellite Markers

Fifteen autosomal dinucleotide microsatellite loci (YWLL08, YWLL29, YWLL36, YWLL38, YWLL40, YWLL43, YWLL46 – Lang et al., 1996, LCA5, LCA19, LCA22, LCA23 – Penedo et al., 1998, LCA65, LCA82 – Penedo et al., 1999, and LGU49, LGU68 – Sarno et al., 2000) were analyzed. Amplification was carried out in a 10 μL reaction volume, containing 50–100 ng of template DNA, 1.5–2.0 mM MgCl2, 0.325 μM of each primer, 0.2 mM dNTP, 1X polymerase chain reaction (PCR) buffer (QIAGEN) and 0.4 U Taq polymerase (QIAGEN). All PCR amplifications were performed in a PE9700 (Perkin Elmer Applied Biosystems) thermal cycler with cycling conditions of: initial denaturation at 95°C for 15 min, followed by 40 cycles of 95°C for 30 s, 52–57°C for 90 s and 72°C for 60 s, and a final extension of 72°C for 30 min. Amplification and genotyping of DNA from fecal samples was repeated two or three times. One primer of each pair was labeled with a fluorescent dye on the 5’-end, and fragments analyzed on an ABI-3100 sequencer (Perkin Elmer Applied Biosystems). Data collection, sizing of bands and analyses were carried out using Genemarker v. 1.70 (SoftGenetics).

We identified fecal samples that came from the same individual by searching for matching microsatellite genotypes using the Excel Microsatellite Toolkit (Park, 2001) and eliminated samples from the study if they showed more than 85% overlap. We also evaluated the existence of null alleles using the program Micro-Checker v. 2.2.3 (Van Oosterhout et al., 2004). ARLEQUIN 3.5.1.2 software (Excoffier and Lischer, 2010) was used to estimate allele frequency, observed heterozygosity (H_O_), and expected heterozygosity (H_E_). The inbreeding coefficient F_IS_ was estimated following Weir and Cockerham (1984) using FSTAT 2.9.4 (Goudet, 2005).

### Genetic Structure and Gene Flow

We used the Bayesian clustering algorithm implemented in STRUCTURE v. 2.3.3 (Pritchard et al., 2000) to group the samples genotyped with microsatellites into *K* clusters. We tested values of *K* between 1 and 23, running STRUCTURE five times for each value of *K*, and using Evanno’s Δ*K* method to determine the most suitable number of clusters (Evanno et al., 2005). STRUCTURE was run using the admixture model and correlated allele frequencies, as recommended for populations that are likely to be similar due to migration or shared ancestry (Falush et al., 2003; Pritchard et al., 2007). 500,000 iterations were used to estimate *K* after a burn-in period of 30,000 iterations.

**Figure 2:**
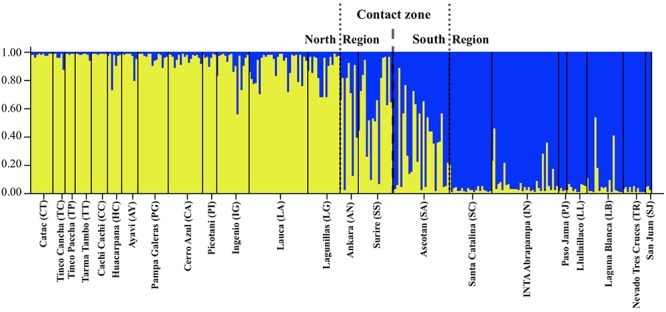
Plot of posterior probability of assignment for 307 vicuñas (vertical lines) to two genetic clusters based on Bayesian analysis of variation at 15 microsatellite loci. Individuals are grouped by locality, and localities are indicated along the horizontal axis. Yellow, Genetic Cluster 1: North group; blue, Genetic Cluster 2: South group.

Based on the STRUCTURE results we found *K* = 2 (Figure 2) to be the most suitable clustering solution (with each cluster corresponding to one subspecies; see results and Figure 2). We used these results to selected the 70 least admixed individuals (35 from northern and 35 from southern localities) to simulate a hybrid population with HybridLab 1.0 (Nielsen et al., 2006). Using these three populations we assessed to which of them each sample in the dataset would be assigned. Furthermore, we also estimated the migration rate between *V. v. mensalis* and *V. v. vicugna* and the hybrid population using BayesAss 3.0 (Wilson and Rannala, 2003; Rannala, 2007). We assessed genetic differentiation between sampling localities using F_ST_ (Weir and Cockerham, 1984) estimated with the microsatellite data and mitochondrial DNA data in ARLEQUIN 3.5.1.2 (Excoffier and Lischer, 2010) with 10,000 permutations to assess significance. We also estimated pairwise population differentiation between sampling localities with Jost’s D in GENODIVE v.2.0b22 (Meirmans and Van Tienderen, 2004), as this method is independent of the amount of within population diversity (Jost, 2008).

### Spatial Autocorrelation Analyses

We tested for spatial autocorrelation in the data at various distance classes using Genalex 6.5 (Peakall and Smouse, 2012). We used Euclidean genetic and geographic distances between pairs of individuals for the species as a whole and at two additional hierarchical levels: (i) separated for males and females to test for sex-biased dispersal and (ii) separated by subspecies. Correlograms of *r*-values were estimated as a function of geographic distance using 200-km fixed distance class bins (shorter distances resulted in few observations per bin whereas longer distances compromised resolution of fine-scale genetic structure). We followed Banks and Peakall (2012) to test for correlogram significance and heterogeneity in allele autocorrelation between sex or subspecies groups using Ω and T2 statistics. One thousand bootstrap permutations were used to estimate the 95% confidence intervals around *r* = 0 (no correlation between genetic variation in individuals in the same bin) and to test if the observed and expected *r-values* were significantly different form each other. Lastly, we tested for the presence of isolation by distance (IBD) using Mantel tests between the matrix of geographic distances between localities and the matrix of Fst estimated for either the mtDNA or the microsatellite loci.

### Migration–Drift Equilibrium

We tested the relative likelihood of a gene flow vs. a genetic drift model using the program 2MOD (Ciofi et al., 1999) (Supplementary Figure 1). Three different datasets were analyzed with 2MOD to test whether the inferred model was affected by the data used. The first dataset consisted of two populations, one made up of the individuals with the highest probability of belonging to *V. v. mensalis* and the other of the individuals with the highest probability of belonging to *V. v. vicugna* based on the STRUCTURE results for *K* = 2 and with a minimum Q threshold of 0.75 indicating the corresponding population ancestry. The second dataset consisted of one population composed of all individuals with a *Q*-value > 0.5 based on the STRUCTURE results for *K* = 2 and the other population made up of all the remaining individuals. The third dataset consisted of the data for each locality separately. Each analysis was run independently three times for 200,000 iterations of the Markov Chain Monte Carlo algorithm with the first 10% of the simulations discarded as burn-in.

### Demographic History

The mtDNA of the northern and southern regions (one group per subspecies) were used to estimate Tajima’s D and Fu’s Fs statistics (Tajima, 1989; Fu, 1997) with 10,000 simulations to assess significance in ARLEQUIN (Schneider and Excoffier, 1999). These analyses were complemented with Bayesian Skyline Plot (BSP) analyses in BEAST run separately for the individuals of each subspecies (one group per subspecies; Bouckaert et al., 2014). BEAST’s Markov Chain Monte Carlo algorithm was run independently three times for 50 million steps discarding the first 10% as burn-in and until ESS values above 200 would be obtained. A substitution rate of 1.2%/million years was used to scale the BSP in years (as in Almathen et al., 2016).

To identify demographic scenarios that explain current diversity patterns in the northern and southern regions, we used the coalescent-based framework implemented in MSVAR v1.3 (Storz and Beaumont, 2002) using microsatellite loci of each sampling locality (see section “Results”). MSVAR estimates the recent effective population size (N0), the ancestral effective population size (Nt), and the time (t) at which the effective population size changed from Nt to N0. Three independent runs of MSVAR were carried out including wide prior distributions of the model parameters and accounting for the possibility that the populations remained stable over time (Nt ∼ N0), that there was a bottleneck (Nt > N0), or a population expansion (Nt < N0). Prior distributions are log-normal distribution parameterized with the mean and standard deviation for each parameter and truncated at zero following Storz and Beaumont (2002) (Supplementary Table 3). Because there is no vicuña specific microsatellite mutation rate available, we used a range of typical vertebrate microsatellite mutation rates varying between 10^-2.5^ and 10^-4.5^ (Weber and Wong, 1993; Di Rienzo et al., 1994; Brohede et al., 2002; Bulut et al., 2009) (Supplementary Table 3). MSVAR was run for a total of 400 million iterations under each demographic model discarding the initial 20% of the MCMC steps as burn-in. The independent runs were used to estimate the mode of the posterior distributions of each parameter (N0, Nt, and t) and their corresponding 90% highest posterior density interval. A generation length of 3 years (Franklin, 1983; González et al., 2006) was used to rescale the t parameter in years. Convergence of the runs was estimated with the Gelman and Rubin’s diagnostic using the CODA library (Plummer et al., 2006) in R (R Development Core Team, 2009).

## Results

### Genetic Diversity

Among the 353 samples genotyped with microsatellites we detected on average 13 alleles/microsatellite. The number of alleles per locus ranged from 5 to 23, and 38 alleles were unique to a single locality. No deviation from H-W equilibrium was found due to an excess of homozygotes, however, a significant excess of heterozygotes (*P* < 0.0151, FDR adjusted critical value) was for various markers in different populations, with ten populations showing no deviation from HWE for any locus. The remaining population showed between one and a maximum of eight locus not in HWE, with an overall mean of 2 loci per locality not in HWE. Overall no linkage disequilibrium was found in the sampling localities, with the exception of 12% o the microsatellite pairwise comparisons in Lauca, and one microsatellite pairwise comparison in Lagunillas, another one in Ascotán, and two in Santa Catalina (however, the loci in these comparisons in the last three populations were not the same; FDR adjusted *P* < 0.00955). Estimates of genetic diversity excluding these loci were not significantly different from those estimated with all loci, thus, we kept these markers for further analyses (Welch corrected *t*-test -*p*-value > 0.05). We found consistently moderate to high levels of genetic diversity (mean expected heterozygosity ranged from 0.45 to 0.78) and high values for allelic richness (mean RA ranged from 2.67 to 7.53) (Table 2) relative to other South American mammals, e.g., andean bear (Ruiz-Garcia et al., 2005), guanacos (Marín et al., 2013a), guigna (Napolitano et al., 2015). In the mtDNA we found 52 variable positions (17.33%), 34 transitions, 7 transversions and one insertion among 385 nucleotides, resulting in 57 haplotypes (*h* = 0.794) among 376 partial sequences of the 5′ end of the control region. Among variable sites, 37 (71.15%) were parsimonious informative. Haplotype (*h*) and nucleotide diversity (π) ranged between 0–0.92 and 0–0.35 (Table 2). GenBank accession for the publicly available data are AY535173–AY535284 and KY420493–KY420569 for the newly generated sequences.

**Table 2:** Genetic diversity indices from 15 microsatellite loci and mtDNA Control Region sequences by localities (defined in Table 1).

	Microsatellites			mtDNA			
Region Localities	*A* ± SD	*Ho* ± SD	*He* ± SD	*n*	*np*	*h* ± SD	π ± SD
**North**	10.73 ± 4.96	0.40 ± 0.16	0.70 ± 0.18	30	26	0.71 ± 0.03	0.010 ± 0.001
CT	2.67 ± 1.03	0.39 ± 0.25	0.45 ± 0.12	3	1	0.60 ± 0.08	0.008 ± 0.001
TC	3.54 ± 1.20	0.39 ± 0.26	0.60 ± 0.26	2	0	0.40 ± 0.11	0.005 ± 0.002
TP	3.33 ± 1.18	0.62 ± 0.34	0.65 ± 0.20	2	1	0.23 ± 0.13	0.001 ± 0.000
TT	3.42 ± 1.56	0.36 ± 0.22	0.56 ± 0.21	3	0	0.71 ± 0.12	0.003 ± 0.001
CC	3.08 ± 1.17	0.44 ± 0.25	0.57 ± 0.14	2	0	0.17 ± 0.13	0.001 ± 0.001
HC	4.23 ± 2.39	0.46 ± 0.28	0.66 ± 0.18	4	1	0.63 ± 0.08	0.009 ± 0.001
AY	3.92 ± 1.32	0.42 ± 0.25	0.63 ± 0.16	2	0	0.57 ± 0.12	0.002 ± 0.000
PG	5.07 ± 2.25	0.50 ± 0.22	0.64 ± 0.21	7	3	0.82 ± 0.05	0.010 ± 0.001
CA	5.21 ± 2.78	0.42 ± 0.27	0.57 ± 0.27	4	1	0.33 ± 0.11	0.001 ± 0.001
PI (captivity)	3.39 ± 1.33	0.48 ± 0.25	0.60 ± 0.18	1	0	0.00 ± 0.00	0.000 ± 0.000
IG	5.73 ± 2.58	0.46 ± 0.23	0.64 ± 0.24	3	0	0.71 ± 0.05	0.003 ± 0.000
LA	7.53 ± 3.58	0.38 ± 0.23	0.68 ± 0.19	10	5	0.73 ± 0.07	0.013 ± 0.004
LG	5.20 ± 2.37	0.42 ± 0.15	0.64 ± 0.19	6	3	0.62 ± 0.14	0.006 ± 0.003
AN	4.53 ± 1.77	0.46 ± 0.24	0.68 ± 0.19	4	1	0.64 ± 0.15	0.004 ± 0.001
SS	6.33 ± 2.52	0.40 ± 0.27	0.71 ± 0.18	8	4	0.89 ± 0.05	0.027 ± 0.002
**South**	11.33 ± 4.50	0.37 ± 0.16	0.74 ± 0.18	31	27	0.84 ± 0.02	0.028 ± 0.001
SA	7.00 ± 3.40	0.35 ± 0.22	0.66 ± 0.23	7	2	0.79 ± 0.08	0.019 ± 0.003
SC	6.40 ± 2.06	0.36 ± 0.19	0.70 ± 0.14	7	3	0.76 ± 0.07	0.026 ± 0.002
IN (captivity)	6.33 ± 2.19	0.34 ± 0.21	0.66 ± 0.19	4	1	0.66 ± 0.05	0.025 ± 0.002
PJ	3.56 ± 1.24	0.44 ± 0.41	0.78 ± 0.16	3	0	0.70 ± 0.22	0.029 ± 0.010
LL	4.36 ± 1.45	0.38 ± 0.23	0.65 ± 0.14	6	1	0.92 ± 0.07	0.031 ± 0.004
LB	5.13 ± 2.23	0.33 ± 0.23	0.66 ± 0.15	9	3	0.83 ± 0.07	0.030 ± 0.002
TR	5.27 ± 1.83	0.49 ± 0.27	0.70 ± 0.19	5	2	0.81 ± 0.12	0.035 ± 0.005
SJ	2.69 ± 0.63	0.49 ± 0.32	0.66 ± 0.14	2	1	0.67 ± 0.31	0.018 ± 0.008

*A, number of alleles per locus; *He*, expected heterozygosity; *Ho*, observed heterozygosity; *n*, number of haplotypes; *np*, number of private haplotypes; *h*, haplotype diversity; π, nucleotide diversity; SD, standard deviation*.

### Genetic Structure and Gene Flow

Results of the STRUCTURE analysis indicated that the best clustering solution was *K* = 2 based on the Δ*K* method (Supplementary Figures 2A,B) with most of the samples from the Northern region being assigned to one cluster and most of the samples from the Southern region being assigned to the other cluster. Of the 353 individuals, 269 had a clear predominant heritage with *Q*-values > 0.75. Of the 173 individuals sampled in the Northern region, 155 presented a predominantly northern genetic background, while 20 presented a mixed South – North origin and 4 had clear South genetic heritage with *Q*-values indicating southern ancestry larger than 0.75 (Table 3). Of the 108 individuals sampled in the Southern Region, 95 presented a predominantly southern genetic background (*Q* > 0.75), while 13 were of mixed origin, and 2 were assigned to the Northern cluster (i.e., their *Q*-values indicating a northern ancestry were > 0.75). Overall, most of the individuals of mixed or incongruent heritage in both clusters were sampled in the localities of AN, SS and SA, which correspond to the contact zone between the North and South regions (Figure 2 and Table 3).

**Table 3:** Percentage of individuals assigned to the North or South genetic clusters with nuclear DNA (*Q* > 75%, STRUCTURE analysis).

Localities		Microsatellites	
	North	Hybrids	South
CT	100.0 (11)	0.0 (0)	0.0 (0)
TC	100.0 (6)	0.0 (0)	0.0 (0)
TP	100.0 (5)	0.0 (0)	0.0 (0)
TT	100.0 (8)	0.0 (0)	0.0 (0)
CC	100.0 (8)	0.0 (0)	0.0 (0)
HC	85.7 (6)	14.3 (1)	0.0 (0)
AY	100.0 (8)	0.0 (0)	0.0 (0)
PG	100.0 (15)	0.0 (0)	0.0 (0)
CA	100.0 (17)	0.0 (0)	0.0 (0)
PI	100.0 (7)	0.0 (0)	0.0 (0)
IG	87.5 (14)	12.5 (2)	0.0 (0)
LA	93.1 (27)	6.9 (2)	0.0 (0)
LG	81.3 (13)	18.7 (3)	0.0 (0)
**AN**	**44.4 (4)**	**33.3 (3)**	**22.2 (2)**
**SS**	**35.3 (6)**	**52.9 (9)**	**11.8 (2)**
**SA**	**7.1 (2)**	**46.4 (13)**	**46.4 (13)**
SC	0.0 (0)	0.0 (0)	100.0 (21)
IN	0.0 (0)	9.1 (3)	90.9 (30)
x PJ	0.0 (0)	0.0 (0)	100.0 (4)
LL	0.0 (0)	0.0 (0)	100.0 (10)
LB	0.0 (0)	11.1 (2)	88.9 (16)
TR	0.0 (0)	0.0 (0)	100.0 (11)
SJ	0.0 (0)	0.0 (0)	100.0 (3)

*The number of individuals from each locality is in parentheses. Localities containing animals from of both clusters, considered to be hybrids (*Q* > 25%), are shaded and in bold*.

The pairwise analysis of divergence between sampling localities showed significant genetic differentiation between ∼58% of the localities pairwise comparisons measured with the *F*_ST_ and (*D)phi-st* (Supplementary Table 1). Significant pairwise differentiation estimated with *F*_ST_ ranged from low (0.037) to high values (0.612), with the greatest divergence observed between the CC and PI localities. *D* pairwise divergence values were larger than the *F*_ST_ values with significant values ranging between 0.19 and 0.95 and the highest divergence observed between the AY and PI localities. Furthermore, we also found a significant negative correlation (-0.56; *P ∼2.2e-16*) in average heterozygosity and pairwise *F*_ST_ between sampling localities suggesting that the observed divergence between localities may be driven by genetic drift rather than the build up of unique mutations present at the different localities (Weeks et al., 2016).

**Figure 3:**
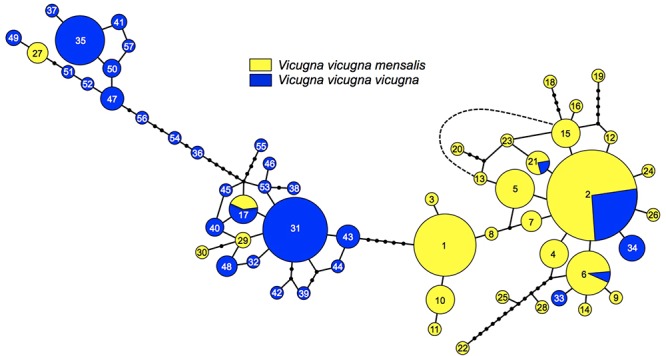
Minimum spanning network representing the relationships among 57 control region haplotypes, numbers represent each haplotype (see Supplementary Table 2). Circle sizes correspond to haplotype frequencies. Branches without circles correspond to one difference between haplotypes, and each small black circle corresponds to one additional mutation. Dashed line represents one mutational step between haplotypes 13 and 16.

When dividing the samples into two groups representing the two clusters identified by STRUCTURE reflecting the two subspecies, the *F*_ST_ including and excluding hybrids were 0.0779 and 0.0998, respectively, and both were significant (*p* < 0.005). A division between the North and South regions was also observed with the haplotype network calculated with the 57 mtDNA Control Region haplotypes (Figure 3). Among these haplotypes 28 were found in *V. v. mensalis* (North) and 25 were found only in *V. v. vicugna* (South), while four where shared between the two subspecies and thus found in both the North and South Regions (haplotype 2, 6, 17, and 21). Additionally, haplotypes 27, 29, and 30 found only on *V. v. mensalis* grouped together with those of *V. v. vicugna*, while haplotypes 33 and 34 found only on *V. v. vicugna* clustered with to the *V. v. mensalis* haplotypes. The 57 haplotypes are connected with a maximum of 48 mutational steps, with most genetic variation localized regionally and the main link between the two geographic regions of the network diverging by five mutations. Consistent with the STRUCTURE results, the haplotypes shared between the two regions occurred in the sampling localities in the contact zone (AN, SS, and SA).

### Spatial Autocorrelation Analysis

**Figure 4:**
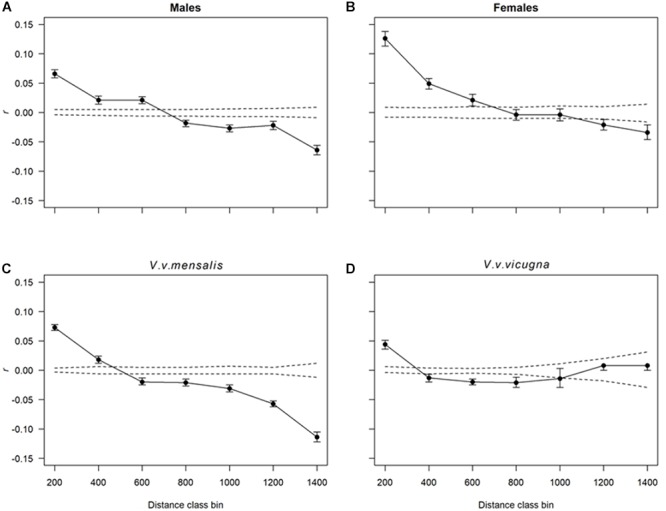
Correlograms showing the combined spatial correlation *r* across transects as a function of distance. Dotted lines correspond to the 95% CI about the null hypothesis of a random distribution of genetic variation over space (i.e., no effect of geographic distance on *r*). Each *r* value has 95% confidence error bars shown. Distance class bins are shown in kilometers. Each plot shows the autocorrelation for distance class sizes of 200 km estimated for **(A)** males, **(B)** females, **(C)** all northern vicuñas (*V. v. mensalis*) and **(D)** southern vicuñas (*V. v. vicugna*).

We found significant spatial autocorrelation among individuals for the species as whole (Ω = 96.9 = 7, *p* < 0.001) and at both hierarchical levels, implying dispersal is limited at the spatial scale, with greater resemblance between individuals at shorter distances and decreasing resemblance as distance increases. Although both males and females presented a spatial autocorrelation pattern indicative of isolation by distance, these were significantly different from each other (Ω = 62.3, *p* < 0.001) with females presenting almost twice as much similarity than males at the smaller distance class (T2 = 63.5, *p* < 0.001 – 200 km distance class). None the less, as geographical distance increases the differences between *r*-values in both sexes almost disappears (Figure 4A,B). We also found significant differences in autocorrelation between subspecies (Ω = 36.2, *p* < 0.001; Figure 4C,D) with the northern vicuña showing a stronger effect of geographic distance on their similarity than in the southern vicuña which shows approximately the same similarity across all distance classes. The analysis of IBD using Mantel tests with the mtDNA data resulted in a significant correlation between the matrix of geographic distances between localities and the Fst between localities (*r* ∼ 0.36, *p*-value = 0.00016). Equivalent results were obtained for this analysis using the microsatellite data (*r* ∼ 0.38, *p*-value = 0.00001).

### Migration–Drift Equilibrium

Results of the coalescent analyses to tests for gene flow + genetic drift vs. a genetic drift only model showed that all simulations support a genetic drift + gene flow model as an explanation of the observed genetic variability (the posterior probability of the model of genetic drift + gene flow was higher than 0.98 for all tests; Supplementary Table 5). This result is the same for all three alternative dataset configurations tested and indicates that vicuña localities have historically not been isolated from each other but, rather, inter connected.

### Demographic History

**Figure 5:**
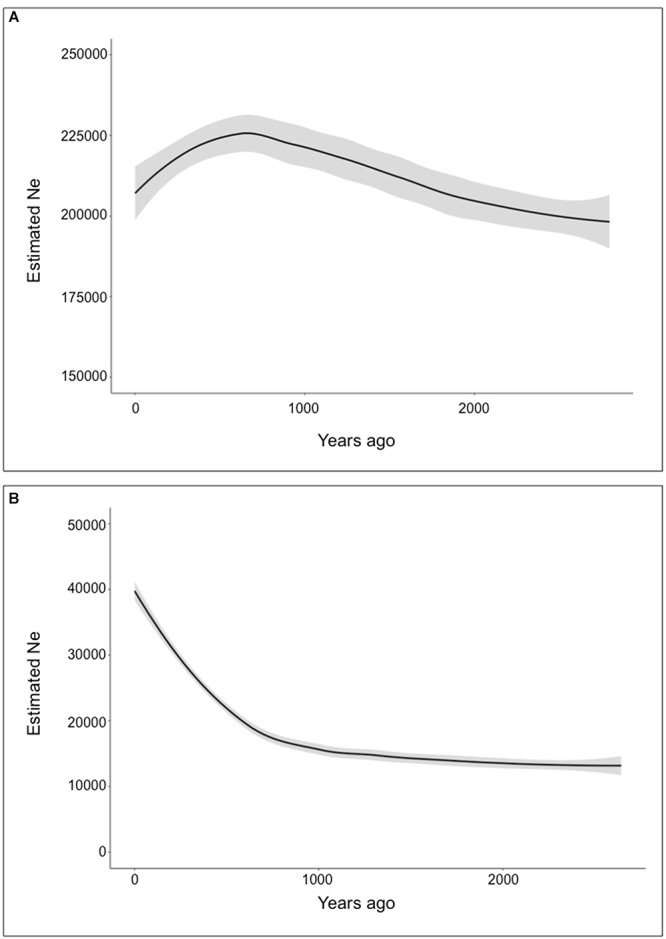
Demographic analysis of *Vicugna vicugna* using the Bayesian Skyline Plot Method. Gray background represents error margins. **(A)** Bayesian Skyline Plot of *Vicugna vicugna mensalis***. (B)** Bayesian Skyline Plot of *Vicugna vicugna vicugna*.

Based on the mtDNA data, the North Region showed negative Fu’s *F*-values (*F*_s_ = -2.3279, *P* < 0.05) and Tajima’s *D* (*D* = -1.7341, *P* > 0.05) consistent with a pattern of population expansion, however, Tajima’s *D* was not significant. For the South region both tests were not significant (*F*_s_ = -0.0863, *P* > 0.10; *D* = 1.2433, *P* > 0.05) suggesting a stable population history. These results are consistent with the presence of a major haplotype in the northern region (Hap2), while in the southern region two distantly related haplotypes occur at moderate to high frequency (Hap31 and 35) with many low frequency haplotypes in between them. The BSP analysis for each subspecies clearly shows a higher effective population size for *V. v. mensalis* (Figure 5A,B). This same analysis also supports a recent population expansion for both *V. v. mensalis* and *V. v. vicugna* starting approximately 3,000 years ago (Figure 5A,B). However, *V. v. mensalis* presents a population decrease starting approximately 800 years ago (Figure 5A).

**Figure 6:**
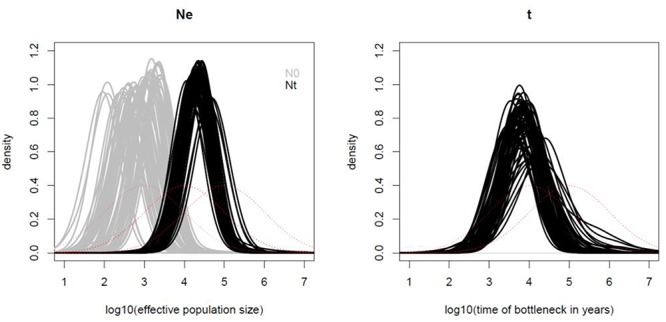
Demographic analysis of *Vicugna vicugna* with MsVar. Panel **(A)** shows the posterior distributions of the current effective population size (gray lines), and ancestral effective population size (black lines). The time of the bottleneck (black lines) is shown in panel **(B)** for each of the three MSVAR replicates for each locality analyzed. The *x*-axis values are in log scale (e.g., 2 means 10^2^). The red lines show the prior distributions.

The demographic analysis using the microsatellite data and MsVar showed consistent results under all three demographic models tested, i.e., (1) no demographic change, (2) bottleneck and (3) population expansion. The three independent runs for each sampling locality presented Gelman and Rubin’s statistics lower than 1.2. In all cases MSVAR detected evidence for major effective population size decline at all localities, consistent with current or recent small census sizes (Figure 6 and Supplementary Table 3). All localities analyzed presented large ancestral effective population sizes on the order of ∼22,000 individuals [with 95% highest posterior density intervals (HPD) between ∼5,000 and ∼100,000; Supplementary Table 3]. Across localities the time of the bottleneck was on average ∼7,600 years before the present (YBP; HPD ∼760–125,000 YBP). Following this event, the effective population size in vicuña reached on average less than 1,000 (HPD ∼200–∼22,000; Supplementary Table 4). Similar ancestral effective population size and bottleneck dates, combined with the results of migration–drift and isolation by distance analyses, suggests that current vicuñas descend from a single large ancestral population that only recently started diverging probably through genetic drift.

## Discussion

Here, we present the most comprehensive analysis to date of genetic variation in vicuña across the species range. The molecular markers used present contrasting patterns regarding vicuña evolutionary history with conflicting evidence regarding the split of the species into two different taxonomic units. For instance, analysis of the mtDNA haplotypes largely supports a split into two vicuña groups, with each group dominated by haplotypes specific to the northern (i.e., *V. v. mensalis*) or the southern (i.e., *V. v. vicugna*) groups, respectively. This apparent differentiation is supported by the divergence analysis that results in a large and significant Φ_CT_ of 0.4. Nevertheless, some haplotypes are shared between the two groups of vicuñas, particularly at localities in the contact zone between 18°S (Ankara, AN) and 21°S (Salar de Ascotan, SA). The data presented here evidences gene flow between the two vicuña types as reflected by haplotype sharing between animals of each type (e.g., haplotypes 2, 6, 17, and 21) deriving from localities at or near the contact zone between the two types (Salar de Ascotan, Santa Catalina, Lauca, Salar de Surire). This is further supported the observation of phenotypically *mensalis* individuals that carry *vicugna* haplotypes (i.e., seven individuals from Salar de Surire carrying haplotypes 27 (four animals), 29 (2 animals), and 30 (1 animal) and conversely, phenotypically *vicugna* individuals that carry *mensalis* haplotypes (i.e., two individuals from Salar de Ascotan carrying haplotype 33 and one carrying haplotype 34, and four individuals from Santa Catalina carrying haplotype 33).

STRUCTURE analysis of the microsatellite data also identifies the presence of two clusters in the dataset (*K* = 2), and supports a contact zone between the two clusters where animals in that region present composite genotypes at intermediate allele frequencies to those observed in more northern or southern vicuñas (Figure 2). Our dataset did not include animals from Bolivia, a region in the contact zone between the two clusters identified here. However, we expect that considering Bolivia’s location, vicuña samples from that part of the range might belong to the hybrid set of genotypes detected here for the contact zone. Such a pattern would explain the lack of taxonomic differentiation previously observed in Bolivia (Sarno et al., 2004). Interestingly, the contact zone between the two vicuña distribution ranges broadly coincides with the area occupied by the Tauka palaeolake which formed after the last glacial maximum and disappeared around 8,500 years ago (Blard et al., 2011). At its maximum, 16,000–14,500 BP, Tauka palaeolake is thought to have covered more than 52,000 km^2^ (i.e., ∼75% the size of Lake Victoria in Africa) including the extant Lake Poopó and the Coipasa and Uyuni saltpans. However, following its disappearance, a narrow area between the mountains to the east of the Atacama Desert and the Uyuni saltpan opened enabling contact between the two vicuña groups, which otherwise would have been separated by the presence of this large lake in the Andean altiplano.

The best clustering solution identified by STRUCTURE with the microsatellite data, while reminiscent of the mtDNA split between the two vicuña subspecies, is more likely the outcome of isolation by distance (see Mantel test results and spatial autocorrelation) where individuals from localities at one end of the range distribution are more likely to resemble each other than individuals on the opposite end of the range. In such a scenario, instead of two populations being present, the data represents a single population with a gradient of intermediate genotypes between two contrasting extremes, as observed in Figure 2. Consistent with such pattern, individuals from the localities in the contact zone between the North and South ranges (i.e., Ankara, Surire and Ascotan), present a variety of genotypes ranging from mostly belonging to one of the two groups to almost presenting 50% ancestry from each group, while animals beyond this region are mostly of one genetic background. This is further supported by the phylogeographic analysis of the DBY gene of the Y chromosome which found no evidence for differences between *V. v. mensalis* y *V. v. vicugna* (Marín et al., 2017).

The change in genetic similarity between animals at increased distance was also observed with the spatial autocorrelation analyses. While splitting the dataset by sex resulted in both groups showing approximately the same isolation by distance pattern, females were more similar to each other at smaller distances (e.g., 200–400 km) than males. This pattern is consistent with females behaving more philopatric than males, with the later leaving their family groups upon becoming yearlings and form non-territorial bachelor groups which frequently have to move because of conflicts with local males with established territories (Koford, 1957; Franklin, 1983; Arzamendia et al., 2018). Yet, both females and males contributed similarly to gene flow at distance classes from 600 to 800 km, suggesting that beyond 800 km the effect of gene flow is limited, as few animals (if any) move that far.

A different pattern of spatial autocorrelation was observed when separately analyzing the northern and southern vicuñas. While northern vicuñas show the same isolation by distance pattern discussed above (Figure 4), the autocorrelogram for the southern vicuña drops quickly between 200 and 400 km, then seems to level off with similar *r*-values across further distances. Northern vicuñas inhabit a geographic range with higher habitat productivity and wider dietary resource availability than southern vicuñas. Thus, while northern vicuñas can find food resources in relative proximity (Franklin, 1982), southern vicuñas need to move over longer distances to find them, thereby increasing the probability of reproduction with animals that otherwise would be too far away (Arzamendia et al., 2018). However, such difference can also be achieved by populations with different levels of genetic variation, where populations with lower genetic diversity will experience a stronger effect of geographic distance if gene flow is low (as in northern vicuñas), while populations with a higher genetic diversity (as in southern vicuñas) need of a stronger reduction in gene flow to result in the same spatial pattern.

Extant vicuña populations are assigned to one of the two recognized vicuña subspecies; however, while these may present some morphological differences possibly reflecting local adaptation, their genetic variation suggests they form two extremes of a genetic continuum. Further evidence about the joint evolution of the two vicuña groups is provided by demographic modeling of the history of the various localities analyzed here, and assessment of whether these localities evolved independently from each other or connected via gene flow. The demographic analysis with MSVAR found that the extant vicuña genetic variation is the outcome of strong bottleneck that occurred ∼7,600 YBP (HPD ∼760–125,000 YBP). However, what is remarkable, is not only that all extant populations seem to have passed through this bottleneck at approximately same time, they all had a very similar ancestral effective population size (i.e., ∼25,000–HPD ∼5,000–100,000). It is likely that a single large vicuña population occupied a wide range across the Andes prior to a relatively recent bottleneck that dramatically reduced the effective population size to less than 1,000 (HPD ∼50–10,000). The main consequence of this event was fragmentation and isolation of previously well connected vicuña populations within their present distribution area (9°S to 29° S) and the small effective population size of pocket populations that survived. This hypothesis is supported by comparison of the model of evolution under genetic drift against a model that also included gene flow and which unambiguously showed that the latter better explains the extant genetic variation. While this analysis does not indicate modern connectivity between these localities, as has been previously suggested for vicuña (Casey et al., 2018), it supports that connection between them has been a major factor in the recent evolutionary history of *V. vicugna*.

The average bottleneck estimate across the vicuña populations is ∼7,600 YBP, but the range of variation from ∼700–125,000 YBP (Supplementary Table 4) reflect the uncertainty associated with estimates like generation length and mutation rate. Thus, while it is tempting to try to associate the inferred bottleneck with a particular event during the South American Holocene, it is safer to assume that it occurred sometime over the last 12,000 years. This period has been marked by dramatic changes across South America including the establishment of human hunters in the Peruvian high Andes ∼9,000 YBP (Aldenderfer, 1999) who, by ∼6,000 YBP, specialized on vicuña and guanaco (Wheeler et al., 1976). Additionally, this period of time included the transition from hunting to herding, with domestication of vicuña ∼6,000–5,500 YBP (Wheeler, 1995). It is possible that any or all of these events contributed to the demographic signal observed here, however, we are not able to pinpoint a single event. While the consistent results obtained across vicuña populations are indicative of the robustness of the genetic signature of the demographic change (Chikhi et al., 2010; Peter et al., 2010), future studies should be carried out using larger datasets (e.g., genome-wide polymorphisms) and other methodologies that are likely to result in narrower confidence intervals of parameters of interest (e.g., approximate Bayesian computation).

### Conservation Implications

#### Management Units

The evolutionary history of extant vicuñas is not at odds with the observation of morphological differences between animals across its range. In fact, it indicates that despite environmental changes during the Late Pleistocene and Holocene, *V. vicugna* maintained its genetic and taxonomic identity through time. Moreover, this identity remained despite the human population expansion in South America (<12,000 YBP) and their specialization in hunting vicuña (and guanaco) (Wheeler et al., 1976), as well as domestication of the vicuña at 6,000–5,500 YBP (Baid and Wheeler, 1993; Wheeler, 1995, 2012). Morphological variation across vicuña is likely to reflect the extensive territory they occupy and the different ecologies they are exposed to. Hence, morphological differences between the northern and southern groups would conform to ecotypes, as in other species, even if those where differences are not obviously reflected by the molecular markers used here (Courtois et al., 2003).

Establishing MUs is difficult because of differentiation by distance and the influence of genetics pools at sampled localities. MU determination depends on geographic areas with independent demographic dynamics between populations, whose individuals present a well-defined genetic structure and low migration rates (Marín et al., 2013a; Sveegaard et al., 2015; Yannic et al., 2016). At the large continental scale, it was not possible for us to identify discrete genetic clusters differentiating localities along the vicuña distribution range in this study, as it was in the study of Peruvian vicuña populations (Wheeler et al., 2001); therefore we propose the use of “continuous” MUs for the species. The main reason for this is that vicuña populations are defined by distance instead of by population discrete fragmentation or geographical barriers. By implementing this approach it is possible to include the spatial correlation information in defining the management area dimensions (e.g., 0–200 km) and protective actions for each locality (e.g., Caughley, 1994), which would enable extending the proposed MUs of Wheeler beyond the only four groups identified in their work (Wheeler et al., 2001).

#### Captive Populations

Two captive populations were included in this study Abra Pampa (Argentina) and Picotani (Peru). These populations present lower genetic variation as measured with both types of molecular markers than their immediate wild neighboring populations (i.e., Santa Catalina and Ingenio, respectively). The Abra Pampa Experimental Station has had captive vicuñas since 1933 (see Mosa, 1987). Although today’s captive population is ∼1,200 individuals (Boswall, 1972; Mosa, 1987; Canedi and Pasini, 1996; Vicuña Convention, 2017) it has been reported that the founding population may have been as few as 22 animals (Canedi and Pasini, 1996). Our results suggest that this population has lower allelic richness and haplotypic diversity indexes than the other localities, probably as a consequence of a founder effect. None the less, the Abra Pampa confinement system has not resulted in a substantial loss of heterozygosity, supporting the hypothesis that a constant but low flow of wild vicuña into the captive herd has taken place (Anello et al., 2016). On the other hand, the Picotani animals have been in captivity since 1997 and they are completely enclosed and there is no breeding with wild vicuña. Our results indicate that the majority of genetic diversity estimators show a reduction in genetic variation in this captive population (e.g., only one mtDNA haplotype was found in comparison to three in neighboring Ingenio) probably due to the founder effect and despite of the larger starting population relative to Abra Pampa. The poor mtDNA genetic variation in captive animals from Picotani is worrying if some management decisions are taken at short-term such as releasing into the wild or translocating for repopulation or productive purposes, therefore a genetic impact assessment is urgently needed for decision support. These results help both setting a basal line for monitoring genetic diversity in these captive populations, but also provide information relevant for the development of an improved long-term captive management strategy at both locations to mitigate the observed loss of genetic variation.

## Conclusion

Here, we present the most extensive genetic analysis of *Vicugna vicugna* to date. These results suggest that the two morphological variants of vicuña, i.e., the northern *V. v. mensalis* and the southern *V. v. vicugna*, were until recently closely interconnected with each other, or probably part of a single large population that passed through a strong bottleneck that left small isolated populations across a vast geographic range. Furthermore, extant vicuña genetic variation is better explained by a model of isolation by distance rather than by two discrete populations. However, given the extent of the vicuña geographic range and variation in the environments therein, it is likely that vicuña populations differ to some extent due to adaptation to local environmental variables. We propose the use of continuous MU for vicuña conservation and that this data serve as a baseline for genetic variation monitoring to avoid further loss of genetic diversity in captivity.

## Ethics Statement

Samples were collected following guidelines of the American Society of Mammalogists (Sikes et al., 2011). Specific permits were required for the Servicio Agrícola y Ganadero, SAG (permit 447, 2002), the Corporación Nacional Forestal, CONAF (permit 6/02, 2002), for granting other collection permits and helping in collecting samples. The animal research oversight committee of Universidad del Bío-Bío had knowledge of sampling plans prior to their approval of the present animal research protocol. All experimental protocols were approved by the Institutional Animal Care and Use Committee of Universidad del Bío-Bío, the methods were carried out in accordance with the approved guidelines.

## Author Contributions

JM developed the ideas and obtained funding for the project. JW, BG, and JM collected the samples. JV, JC, RR, NA, and AC conducted the DNA analyses. AC, AA, DG-U, VV, and PO-tW analyzed the data. JM, BG, DG-U, JW, and PO-tW wrote the manuscript. All authors read, commented on and approved the final manuscript.

## Conflict of Interest Statement

The authors declare that the research was conducted in the absence of any commercial or financial relationships that could be construed as a potential conflict of interest.
