# Supplementary material for: Phylogeography and Population Genetics of Vicugna vicugna: Evolution in the Arid Andean High Plateau
Source: Front Genet. 2019 Jun 6;10:445. doi: 10.3389/fgene.2019.00445 (PMC6562099; doi:10.3389/fgene.2019.00445)

**Additional Information**

**Supplementary Table 1.** Pairwise *F_ST_* and *D(phi-st)* values between vicuña sampling localities. *F_ST_* values are shown above the diagonal and *D(phi-st)* values below the diagonal. Significant values after FDR correction are highlighted in bold.

|  | **CT** | **TC** | **TP** | **TT** | **CC** | **HC** | **AY** | **PG** | **CA** | **PI** | **IG** | **LA** | **LG** | **AN** | **SS** | **SA** | **SC** | **IN** | **PJ** | **LL** | **LB** | **TR** | **SJ** |
| --- | --- | --- | --- | --- | --- | --- | --- | --- | --- | --- | --- | --- | --- | --- | --- | --- | --- | --- | --- | --- | --- | --- | --- |
| **CT** | - | **0.276** | **0.496** | **0.386** | **0.517** | **0.319** | **0.382** | **0.318** | **0.309** | **0.498** | **0.304** | **0.266** | **0.241** | **0.359** | **0.278** | **0.237** | **0.328** | **0.356** | **0.372** | **0.276** | **0.192** | **0.245** | 0.128 |
| **TC** | 0.068 | - | **0.329** | **0.269** | **0.404** | **0.145** | **0.178** | **0.132** | **0.192** | **0.406** | **0.169** | **0.128** | **0.156** | **0.228** | **0.148** | **0.118** | **0.188** | **0.240** | 0.150 | 0.139 | 0.101 | 0.099 | 0.096 |
| **TP** | **0.486** | 0.096 | - | 0.031 | -0.004 | **0.305** | 0.057 | **0.177** | 0.042 | **0.523** | **0.237** | 0.103 | **0.161** | **0.261** | **0.205** | **0.190** | **0.241** | **0.266** | 0.336 | **0.298** | **0.272** | **0.244** | 0.358 |
| **TT** | 0.356 | 0.046 | 0.188 | - | 0.020 | **0.235** | 0.066 | **0.166** | 0.065 | **0.472** | **0.216** | **0.119** | **0.161** | **0.265** | **0.158** | **0.187** | **0.261** | **0.298** | 0.305 | **0.285** | **0.254** | **0.226** | 0.235 |
| **CC** | **0.484** | 0.096 | 0.036 | 0.166 | - | **0.376** | 0.111 | **0.282** | **0.148** | **0.612** | **0.348** | **0.207** | **0.264** | **0.398** | **0.282** | **0.287** | **0.358** | **0.391** | **0.467** | **0.416** | **0.359** | **0.350** | **0.414** |
| **HC** | 0.007 | 0.104 | **0.457** | **0.344** | **0.440** | - | 0.121 | **0.122** | **0.105** | **0.344** | 0.084 | **0.105** | 0.046 | **0.138** | 0.063 | 0.066 | **0.159** | **0.206** | 0.142 | **0.204** | **0.186** | 0.091 | 0.156 |
| **AY** | **0.339** | **0.435** | **0.925** | **0.842** | **0.933** | **0.323** | - | **0.146** | 0.060 | **0.436** | **0.172** | **0.111** | **0.121** | **0.223** | **0.138** | **0.155** | **0.219** | **0.269** | **0.250** | **0.268** | **0.214** | **0.175** | 0.187 |
| **PG** | -0.012 | 0.017 | **0.287** | 0.196 | **0.281** | 0.012 | 0.331 | - | 0.042 | **0.174** | 0.031 | 0.006 | 0.023 | 0.056 | 0.031 | 0.041 | **0.107** | **0.131** | 0.075 | **0.099** | **0.140** | 0.048 | **0.207** |
| **CA** | **0.516** | **0.123** | 0.039 | 0.078 | -0.011 | **0.490** | **0.898** | **0.316** | - | **0.240** | 0.041 | 0.020 | 0.020 | 0.052 | 0.038 | **0.059** | **0.124** | **0.126** | 0.111 | **0.145** | **0.148** | **0.082** | **0.167** |
| **PI** | **0.473** | 0.083 | 0.025 | 0.181 | -0.016 | **0.439** | **0.950** | 0.273 | -0.017 | - | **0.197** | **0.158** | **0.164** | 0.139 | **0.221** | **0.170** | **0.221** | **0.230** | 0.250 | **0.180** | **0.302** | **0.192** | **0.489** |
| **IG** | **0.395** | 0.107 | **0.267** | -0.078 | **0.243** | **0.387** | **0.815** | **0.242** | **0.184** | 0.240 | - | 0.015 | -0.004 | 0.006 | 0.005 | -0.018 | 0.017 | 0.043 | 0.055 | **0.093** | **0.114** | 0.025 | 0.187 |
| **LA** | **0.197** | 0.034 | 0.037 | 0.000 | 0.008 | **0.195** | **0.502** | **0.119** | 0.041 | 0.008 | 0.064 | - | 0.027 | 0.039 | 0.020 | **0.037** | **0.093** | **0.094** | 0.071 | 0.063 | **0.090** | 0.040 | **0.163** |
| **LG** | **0.345** | 0.062 | 0.025 | -0.002 | -0.010 | **0.343** | **0.736** | **0.211** | 0.021 | -0.016 | 0.094 | -0.011 | - | 0.015 | 0.002 | 0.016 | 0.082 | 0.105 | 0.066 | 0.094 | 0.114 | 0.019 | 0.117 |
| **AN** | **0.330** | 0.036 | 0.104 | 0.051 | 0.053 | **0.313** | **0.785** | 0.175 | 0.054 | 0.063 | 0.145 | -0.013 | 0.002 | - | 0.042 | 0.011 | 0.052 | 0.021 | 0.020 | 0.084 | **0.126** | 0.035 | 0.234 |
| **SS** | **0.290** | **0.238** | **0.409** | **0.306** | **0.368** | **0.241** | **0.348** | **0.276** | **0.469** | **0.359** | **0.378** | **0.296** | **0.346** | **0.308** | - | -0.003 | 0.049 | **0.085** | 0.085 | 0.091 | **0.090** | 0.021 | 0.155 |
| **SA** | 0.209 | 0.104 | 0.273 | 0.186 | 0.240 | 0.166 | **0.404** | **0.170** | **0.326** | 0.235 | **0.256** | **0.151** | 0.213 | 0.180 | 0.055 | - | 0.039 | **0.047** | 0.041 | **0.081** | **0.086** | 0.020 | 0.130 |
| **SC** | **0.215** | **0.187** | **0.360** | **0.274** | **0.333** | **0.200** | **0.288** | **0.230** | **0.419** | **0.320** | **0.338** | **0.278** | **0.314** | **0.277** | 0.006 | 0.034 | - | 0.040 | 0.106 | **0.100** | **0.124** | **0.076** | 0.179 |
| **IN** | **0.361** | **0.355** | **0.476** | **0.425** | **0.461** | **0.346** | **0.371** | **0.382** | **0.512** | **0.455** | **0.460** | **0.432** | **0.450** | **0.427** | 0.080 | **0.200** | 0.036 | - | 0.055 | **0.104** | **0.116** | **0.100** | **0.242** |
| **PJ** | - | - | - | - | - | - | - | - | - | - | - | - | - | - | - | - | - | - | - | 0.089 | 0.122 | 0.055 | 0.201 |
| **LL** | **0.434** | **0.405** | **0.623** | **0.488** | **0.580** | **0.421** | **0.412** | **0.448** | **0.689** | **0.559** | **0.579** | **0.486** | **0.545** | **0.498** | 0.025 | 0.226 | 0.027 | -0.021 | - | - | 0.042 | 0.036 | 0.151 |
| **LB** | **0.240** | **0.213** | **0.383** | 0.292 | **0.353** | **0.233** | **0.263** | **0.250** | **0.443** | **0.338** | **0.359** | **0.308** | **0.335** | **0.296** | -0.003 | 0.088 | -0.025 | 0.029 | - | -0.040 | - | 0.039 | 0.072 |
| **TR** | **0.248** | 0.208 | **0.433** | 0.294 | **0.390** | **0.251** | 0.269 | **0.260** | **0.518** | 0.365 | **0.397** | **0.311** | **0.356** | 0.308 | 0.009 | 0.102 | -0.025 | 0.043 | - | -0.050 | -0.065 | - | 0.096 |
| **SJ** | **0.684** | **0.623** | **0.917** | **0.825** | **0.906** | **0.636** | 0.756 | **0.663** | **0.921** | **0.906** | **0.856** | **0.655** | **0.800** | **0.804** | 0.142 | **0.392** | 0.096 | -0.051 | - | -0.068 | 0.043 | 0.031 | - |

Supplementary Table 2*.* Distribution of the 57 control region haplotypes observed in 353 vicuñas from 23 localities. The vertical numbers indicate the position of polymorphic sites relative to haplotype 1. For each haplotype, taxon (m, *V. v. mensalis*; v, *V. v. vicugna*), number of individuals (n) between parenthesis and localities.

Haplotype 1111111122222222 Taxon(n) Localities

1111111122233333344445556666667891167899911112379

2691234567948901367813464890457892405694101501789008

Hap_1 TGAAACCGGCAGTTAATTACTGTCGTTTTCAGCGATCTCTCCATCTGTCCTT m(28) CT, TC, HC, AY, PG

Hap_2 .......A..................................G..C..T... m(111), v(34) CT, TT, CC, TP, TC,

PG, CA, PI, LA, LG,

AN, SS, SA, PJ, SC,

IN, LL, LB, TR

Hap_3 A................................................... m(1) CT

Hap_4 .......A...................C..............G..C..T... m(7) TT, IG

Hap_5 .......A.....................................C..T... m(11) TT, CA, IG, LG

Hap_6 .......A..................................G..CA.T... m(17), v(1) CC, HC, PG, LA, SS,

SA

Hap_7 .......A..................................G.....T... m(2) TP

Hap_8 ................................................T... m(1) HC

Hap_9 .......A..................................G..CACT... m(2) HC

Hap_10 .......................................C............ m(7) AY, PG

Hap_11 .......................................C..........C. m(1) PG

Hap_12 .....G.A..................................G..C..T... m(1) PG

Hap_13 .......A.....................................C...... m(1) PG

Hap_14 .......A.......G..........................G..CA.T... m(1) CA

Hap_15 .......A..................................G..C...... m(7) CA, LA, LG, SS

Hap_16 .......A................................T.G..C...... m(2) LG

Hap_17 .............C............C....AT.G...........ACT... m(3), v(4) LA, SS, SA

Hap_18 ....C.A.A.................................G..C...... m(1) LA

Hap_19 ...G.G.A.........A...TA.A.................G..C...... m(1) LA

Hap_20 .A.....A...............A............G.T......C...... m(1) LA

Hap_21 .A.....A..................................G..C..T... m(4), v(1) LA, LG, SC

Hap_22 .A........G...G.C.G.....AA.C...A.A.C.........CA.TA.. m(2) LA

Hap_23 .A.....A..................................G..C...... m(1) LA

Hap_24 .......A.................................TG..C..T... m(1) LG

Hap_25 ..........G........T....A..C...A.............CA.T... m(1) LG

Hap_26 .......A...A............A.................G..C..T... m(1) LG

Hap_27 ..G.....A.G.CC............C....AT......C...C..A.T... m(4) SS

Hap_28 ..........G................C...A..........G..CA.T.C. m(1) SS

Hap_29 .............C............C....AT.G..........CAC.... m(2) SS

Hap_30 .............C..........A.C....AT.G........C.CAC.... m(1) SS

Hap_31 .............C............C....AT.G...........AC.... v(27) SA, SC, IN, LL, LB

Hap_32 .............C............C....AT.G....C......AC.... v(1) SA

Hap_33 .......A............C.....................G..CA.T... v(1) SA

Hap_34 .......A...........T......................G..C..T... v(6) SA, SC

Hap_35 ..G.....A.G.CC............C.C.GAT..........C....T... v(15) SC, IN

Hap_36 .A...........C............C.C.GAT..........C....T... v(1) SC

Hap_37 ..G.....A.G.C.............C.C.GAT..........C....T... v(1) SC

Hap_38 .A...........C............C....AT.G...........A...C. v(1) SC

Hap_39 ...........A.C...A....A...C....AT.G...........AC.... v(1) SC

Hap_40 .............C............C....AT.G..........CACT... v(3) SC

Hap_41 ..G.....A.G.CC............C.C.GAT...............T... v(2) SC

Hap_42 .........A...C...A....A...C....AT.G...........AC.... v(1) SC

Hap_43 .............C............C....AT.............AC.... v(5) IN, TR, SJ

Hap_44 ...........A.C............C....AT.............AC.... v(1) SC

Hap_45 .A...........C............C....AT.G..........CACT... v(1) IN

Hap_46 .A...........C............C....AT.G...........AC...C v(1) IN

Hap_47 ..G.....A.G.CC............C.C.GAT......C...C........ v(5) SC, LB, TR

Hap_48 .............C............C....AT.G....C.....CAC.... v(4) LL, LB

Hap_49 ..G.....A.G.CC............C....AT..........C..A.T... v(2) LL, LB

Hap_50 ..G.....A.G.CC............C.C.GAT..........C........ v(3) LL, LB

Hap_51 ..G.....A.G.CC............C...GAT......C...C....T... v(1) LL

Hap_52 ..G.....A.G.CC............C...GAT......C...C........ v(1) LB

Hap_53 .A...........C............C....AT.G...........AC.... v(1) LB

Hap_54 .A........G..C............C.C.GAT......C...C....T... v(1) LB

Hap_55 .A...........C............C..T.AT.G..AT.....T.ACT... v(1) TR

Hap_56 .A......A.G.CC............C.C.GAT......C...C........ v(1) TR

Hap_57 ..G.....A.G.CC............C.C.GAT................... v(1) SJ

**Supplementary Table 3.** Prior distributions of the demographic parameters inferred using MsVar. The demographic parameters are the estimated current effective population size (No), the ancestral effective population size (Nt), the mutation rate (mu), and the time of the bottleneck in years (t). Prior distributions are log-normal distributions parameterised with the mean and standard deviation (SD) for each parameter and truncated at zero following Storz and Beaumont (2002). The values on the table correspond to the priors of each of the parameters shown. LB = low boundary (mean – SD), HB = high boundary (mean +SD). The hyperprior mean of means and variance of means had the same values as the mean and range in the prior, respectively, and the mean of variances and variance of variances were left as default.

|  | No | | | Nt | | | mu | | | t | | |
| --- | --- | --- | --- | --- | --- | --- | --- | --- | --- | --- | --- | --- |
| Scenario | LB | mean | HB | LB | mean | HB | LB | mean | HB | LB | mean | HB |
| Bottleneck | 100 | 1,000 | 10,000 | 10,000 | 100,000 | 1,000,000 | 0.000032 | 0.00032 | 0.0032 | 10,000 | 100,000 | 1,000,000 |
| Stable | 1,000 | 10,000 | 100,000 | 1,000 | 10,000 | 100,000 | 0.000032 | 0.00032 | 0.0032 | 10,000 | 100,000 | 1,000,000 |
| Expansion | 10,000 | 100,000 | 1,000,000 | 1,000 | 10,000 | 100,000 | 0.000032 | 0.00032 | 0.0032 | 1,000 | 10,000 | 100,000 |

**Supplementary Table 4.** Mode and 95% highest posterior credibility estimates of the demographic parameters inferred using MsVar across the three independent runs of the software carried out. The demographic parameters are the estimated current effective population size (No), the ancestral effective population size (Nt), the mutation rate (mu), and the time of the bottleneck in years (t). For each locality the estimate of the mode, the lower and higher bounds of the 95% highest posterior density (HPDLow and HPDHigh, respectively) across the three independent MsVar runs are shown. Numbers in the table are in years for t, mutation rate per generation for mu, and diploid effective population size for No and Nt.

| Localities | No | | | Nt | | | mu | | | t | | |
| --- | --- | --- | --- | --- | --- | --- | --- | --- | --- | --- | --- | --- |
|  | mode | HPDLow | HPDHigh | mode | HPDLow | HPDHigh | mode | HPDLow | HPDHigh | mode | HPDLow | HPDHigh |
| CT | 115 | 17 | 479 | 50119 | 7244 | 251189 | 0.00023 | 0.00008 | 0.00062 | 3715 | 759 | 25119 |
| TC | 316 | 68 | 1820 | 26303 | 4786 | 120226 | 0.00022 | 0.00008 | 0.00065 | 7586 | 676 | 48978 |
| TP | 234 | 48 | 1318 | 30903 | 5754 | 125893 | 0.00020 | 0.00008 | 0.00063 | 5370 | 891 | 34674 |
| CC | 355 | 69 | 1738 | 20893 | 2754 | 117490 | 0.00023 | 0.00008 | 0.00066 | 8710 | 977 | 74131 |
| HC | 661 | 93 | 5012 | 23442 | 3715 | 102329 | 0.00021 | 0.00008 | 0.00069 | 4467 | 380 | 91201 |
| AY | 468 | 89 | 2399 | 20893 | 6310 | 117490 | 0.00022 | 0.00008 | 0.00068 | 5623 | 871 | 38019 |
| PG | 1000 | 191 | 3981 | 20893 | 4677 | 87096 | 0.00024 | 0.00009 | 0.00069 | 6761 | 891 | 45709 |
| CA | 676 | 78 | 2884 | 23988 | 5754 | 91201 | 0.00024 | 0.00008 | 0.00066 | 3631 | 468 | 24547 |
| PI | 407 | 45 | 2692 | 10233 | 2884 | 60256 | 0.00021 | 0.00007 | 0.00060 | 3162 | 363 | 42658 |
| IG | 2239 | 550 | 10471 | 14454 | 2884 | 107152 | 0.00022 | 0.00009 | 0.00078 | 19498 | 550 | 831764 |
| LA | 1738 | 363 | 6457 | 22909 | 6761 | 95499 | 0.00026 | 0.00009 | 0.00071 | 7244 | 1047 | 44668 |
| LG | 1413 | 263 | 5370 | 20893 | 5012 | 87096 | 0.00043 | 0.00009 | 0.00069 | 6918 | 912 | 72444 |
| AN | 1096 | 151 | 5754 | 26915 | 6310 | 102329 | 0.00025 | 0.00009 | 0.00068 | 5370 | 676 | 39811 |
| SS | 1660 | 302 | 9550 | 16218 | 3802 | 83176 | 0.00028 | 0.00009 | 0.00076 | 12303 | 339 | 239883 |
| SA | 2692 | 447 | 9550 | 17378 | 3311 | 117490 | 0.00028 | 0.00009 | 0.00076 | 7943 | 646 | 562341 |
| IN | 1349 | 316 | 5370 | 19498 | 5754 | 83176 | 0.00022 | 0.00009 | 0.00071 | 7079 | 1148 | 57544 |
| SC | 1862 | 525 | 7943 | 25119 | 6166 | 89125 | 0.00025 | 0.00009 | 0.00074 | 9550 | 1514 | 72444 |
| LL | 447 | 62 | 2754 | 19498 | 5623 | 81283 | 0.00022 | 0.00008 | 0.00065 | 3388 | 398 | 26303 |
| LB | 741 | 117 | 2754 | 24547 | 4677 | 93325 | 0.00022 | 0.00008 | 0.00066 | 5754 | 871 | 41687 |
| TR | 2344 | 282 | 8913 | 15849 | 4571 | 91201 | 0 | 0 | 0 | 12882 | 741 | 134896 |
| SJ | 724 | 115 | 3802 | 19055 | 3236 | 100000 | 0 | 0 | 0 | 11749 | 871 | 112202 |

**Supplementary Table 5.** Posterior probability distributions for the model of gene flow + genetic drift and the only genetic drift model. Results are shown for the analysis using i) only two populations based on the Q threshold of 0.75 (NS), ii) using a Q threshold of 0.5 (NMS), and iii) each locality (POPS).

| Model | Run | gene flow + genetic drift | genetic drift |
| --- | --- | --- | --- |
| NS | 1 | 100.0000 % | 0.0000 % |
|  | 2 | 99.9975 % | 0.0025 % |
|  | 3 | 99.9950 % | 0.0050 % |
| NMS | 1 | 100.0000 % | 0.0000 % |
|  | 2 | 100.0000 % | 0.0000 % |
|  | 3 | 100.0000 % | 0.0000 % |
| POPS | 1 | 98.6500 % | 1.3500 % |
|  | 2 | 99.8800 % | 0.1200 % |
|  | 3 | 98.9800 % | 1.0200 % |

**Supplementary Figure 1.** Alternative demographic models tested with 2mod. **A**: demographic model of population divergence only via genetic drift, and **B**: demographic model of population divergence via genetic drift + gene flow.


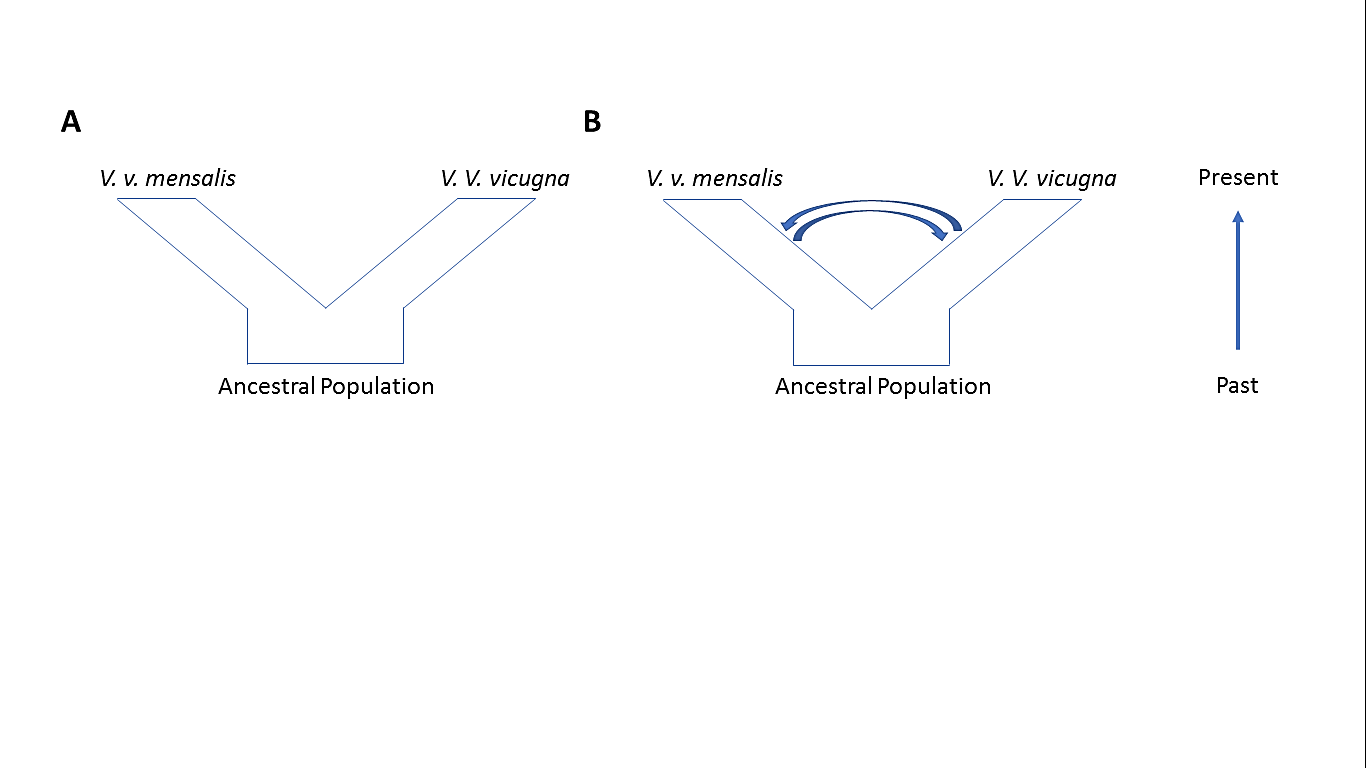


**Supplementary Figure 2.** Support for defining the number of vicuña populations based on the microsatellite data set. **A**: Δ*K* (Delta K = mean(⏐L’’(K)⏐) /sd(L(K))) following Evanno *et al* (2005) as a function of *K*; **B**: Mean estimated values of the logarithm of the probability of each clustering solution (*K*) estimated with STRUCTURE. Each circle has the standard deviation of mean estimates shown as a vertical line.


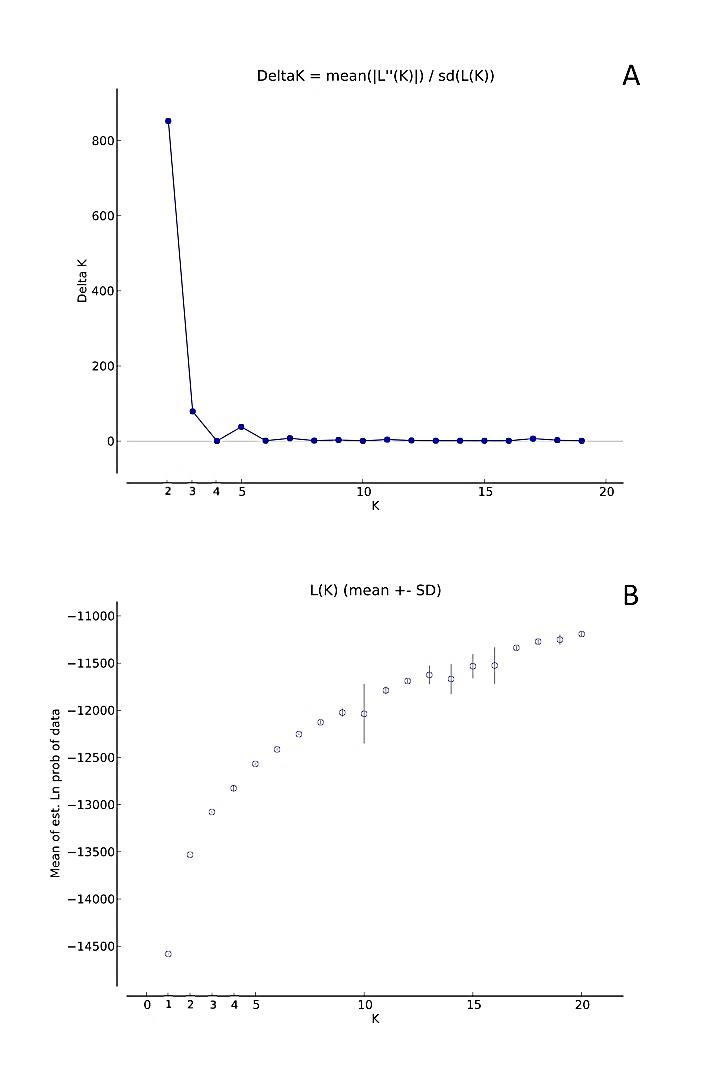

Supplement: Supplementary file 1 [file Data_Sheet_1.docx]
